# Hypospadias algorithm: The way to propose

**Published:** 2010

**Authors:** Wagih Mommtaz Ghnnam

**Affiliations:** Mansoura Faculty of Medicine, General Surgery Department, Mansoura University, Egypt

Tubularized incised plate repair is based on an old principle of urethral plate tubularization, also known as the Thiersch-Duplay procedure. Although a good concept, its main drawback was the limitation imposed by the width of the urethral plate. Snodgrass popularized the concept of urethral plate incision with subsequent tubularization and secondary dorsal healing for primary hypospadias repair. This relatively simple yet elegant and effective procedure has gained widespread acceptance, currently being recognized as the surgical technique of choice for distal and other types of hypospadias repair.[1‐3] In the present study, it is a good effort from the authors to propose the algorithm for hypospadias repair; however, to my mind, it cannot be postulated according to these small number of cases along with no comparison with different techniques and choices.

Decision making in hypospadias repair is no longer determined by meatal location, as in the past, but by severity of penile curvature and appearance of the incised urethral plate. Severe curvature, requiring plate transection or an "unhealthy" incised plate are uncommonly encountered.

A treatment Algorithm must consider the following:

- Differentiation between functionally necessary and aesthetically feasible operative procedures
- Establishing therapeutic objectives
- Pre-operative hormonal treatment
- Age at surgery
- Penile curvature
- Preservation of the well-vascularised urethral plate
- Re-do hypospadias repairs
- Urethral reconstruction
- Urine drainage and wound dressing
- Adolescent satisfaction with penis size
- Rate of fistula formation

The second thing is the rate of fistulae. Although it is comparable to the literature, they do not favour use of a two-layer coverage and deny that Snodgrass has recommended two-layer neourethra closure to decrease fistula formation in all types of hypospadias defects. Several factors may influence fistula formation, such as surgical technique, delicate tissue handling, patient age, type of hypospadias defect, surgeon's experience, water-proof urethroplasty coverage and concomitant foreskin reconstruction.

The meatal stenosis rate varies from 0 to 17%, and Snodgrass has reported meatal stenosis rates below 1%. This has been a controversial topic and considered to be possibly related to the surgical technique. In the present study, meatal stenosis was high (8.8%), with no explanation for this high rate.
